# On the Evolution and Optimality of Mood States

**DOI:** 10.3390/bs3030501

**Published:** 2013-08-26

**Authors:** Pete C. Trimmer, Elizabeth S. Paul, Mike T. Mendl, John M. McNamara, Alasdair I. Houston

**Affiliations:** 1 School of Biological Sciences, University of Bristol, Woodland Road, Bristol BS8 1UG, UK; E-Mail: a.i.houston@bristol.ac.uk; 2 Division of Animal Health and Husbandry, Langford House, Langford, Bristol BS40 5DU, UK; E-Mails: e.paul@bristol.ac.uk (E.S.P.); mike.mendl@bristol.ac.uk (J.M.M.); 3 School of Mathematics, University of Bristol, University Walk, Bristol BS8 1TW, UK; E-Mail: john.mcnamara@bristol.ac.uk

**Keywords:** emotion, circumplex, mood, drift diffusion model, optimal decision making, core affect

## Abstract

Moods can be regarded as fluctuating dispositions to make positive and negative evaluations. Developing an evolutionary approach to mood as an adaptive process, we consider the structure and function of such states in guiding behavioural decisions regarding the acquisition of resources and the avoidance of harm in different circumstances. We use a drift diffusion model of decision making to consider the information required by individuals to optimise decisions between two alternatives, such as whether to approach or withdraw from a stimulus that may be life enhancing or life threatening. We show that two dimensions of variation (expectation and preparedness) are sufficient for such optimal decisions to be made. These two dispositional dimensions enable individuals to maximize the overall benefits of behavioural decisions by modulating both the choice made (e.g., approach/withdraw) and decision speed. Such a structure is compatible with circumplex models of subjectively experienced mood and core affect, and provides testable hypotheses concerning the relationships that occur between valence and arousal components of mood in differing ecological niches. The paper is therefore a useful step toward being able to predict moods (and the effect of moods) using an optimality approach.

*To the cognition of the brain must be added the experience of the soul.*

—Arnold Bennett

## 1. Introduction

In humans, decisions are not just influenced by current requirements and circumstances but also by emotional states, which, in turn, are moderated by past experience. This use of moods (or emotions) as information in decision-making can sometimes be problematic, leading to errors of judgement [1,2] but on many occasions, it may be a valuable heuristic tool (the so called “how do I feel about it?” heuristic), particularly when choices need to be made in the absence of complete information (e.g., [1,3,4]). Facets of this phenomenon have also been observed across a variety of species, suggesting an extensive evolutionary history (e.g., see [5,6]). To date, economic decision theory and theoretical behavioural ecology have often side-stepped the effect of (or need to model) the influence of moods on decision-making. To understand decision-making systems more thoroughly, a suitable model of mood states and a mechanistic representation of how such states influence decisions are required. In this paper, we take a step in this direction by showing how a well-known model of optimal decision-making, the drift diffusion model, can be related to a simple representation of emotional mood or *affective state* that is very similar to “circumplex” representations of human emotional experiences (e.g., see [7,8,9,10,11,12,13,14,15,16,17,18,19]).

In evolutionary terms, discrete emotions such as fear, anger, or joy can be thought of as specialized, multifaceted, functional schema – coordinated sets of processes which allow appropriate and efficient behavioural responses to be made to a wide range of fitness threatening and fitness enhancing stimuli or events (e.g., [20,21]). Ongoing mood states, on the other hand, which are less clearly triggered by individual stimuli, have proven more of a puzzle for those seeking functional explanations. Contemporary accounts of human mood offer two related conceptualizations. First, moods can be thought of as consciously experienced, free-floating feeling states. These states usually change gradually over time, with the feelings associated with briefer, discrete emotions overlaying and interacting with them (e.g., a low mood may accentuate a loss-associated sadness, while a happy mood may be heightened further by a joyful event). However, felt moods can also sometimes continue for considerable durations, as in chronic disorders such as depression and generalised anxiety disorder (American Psychiatric Association, 2000). Second, moods can be thought of as dispositions. These are tendencies to make positive or negative evaluations, and thereby bias behavioural decisions (e.g., safe or dangerous leading to approach or withdraw, edible or inedible leading to consume or avoid), particularly in circumstances where information concerning the expected values of the possible outcomes of such decisions is uncertain [6,22,23,24]. For example, individuals in negative or anxious moods tend to avoid potentially dangerous situations more readily, while those in happy moods tend to interpret ambiguous stimuli and events more positively (e.g., [25]). Like the feeling states that often accompany them, these dispositions may change gradually over time, or become rigid and long-lasting, as in pathological mood disorders. 

Although they are not mutually exclusive, it is the second of these conceptualizations that has become the focus of recent comparative and evolutionary accounts of mood (e.g., [6,23,24,26,27]). By viewing moods as decision-relevant dispositions rather than exclusively as feelings, philosophical debates concerning whether and in what ways the conscious, subjective component might influence decision-making can be put to one side [6,28]. The resulting process models of moods or “affective states” (a term that can be used to describe moods or emotions that does not assume the presence of a subjectively experienced component) are therefore generalizable across many types of animals, and allow testable hypotheses to be constructed which predict variation both within and between species according to differences in their life histories.

We consider the paradigmatic problem of deciding whether to approach a potential food resource (in the form of prey), or withdraw to avoid the risk of being attacked (by a predator). In doing so, we identify circumstances under which mood-like dispositions (*i.e.* affective states) can enable optimal decision-making. We also consider how such functional models of mood resemble contemporary “circumplex” models of human subjective experience (e.g., [12,13,19]) and show that the two approaches can be fully compatible in the context of approach-avoidance decisions.

## 2. Representing Mood State

Many attempts have been made to represent and inter-relate emotional mood states (e.g., see Figure 2.1 of [29]; Figure 1 of [30]). In this paper, we are particularly interested in the finding that moods can be described as corresponding to positions in two-dimensional space and the suggestion that the resulting affective states may be relevant to a wide range of species, not just humans [6]. 

Recent evolutionary considerations of mood have attempted to delineate its basic structure—to establish the fundamental dimensions, which underlie the role of affective state in determining variation in behavioural decisions. In this respect, functional approaches have mirrored those of psychologists studying the structure of moods and emotions as consciously experienced feeling states (e.g., circumplex models—[12,19]), although with somewhat different conclusions. For example, Carver and Scheier [31] (see also [8,16,32]) proposed two bipolar systems of mood relating to the management of behaviour: one that predominantly controls approach to fitness enhancing resources, and another whose focus is the avoidance of and withdrawal from threat. Although these conceptualizations were originally used to describe only consciously felt moods in humans, other researchers (e.g., [33,34]) have hypothesised similar structures, which need not be human-specific (nor consciously experienced). Adaptive behavioural dispositions may exist in a wide range of species to potentiate acquisition of resources in resource rich environments and avoidance or withdrawal in threatening or dangerous environments. For example, assuming that the rate of predation threats in an environment shows a degree of consistency across time (*i.e.* moderate correlation between one time point and the next [23]), an individual that has repeatedly suffered threats of attack may be wise to assume that it is living in the sort of environment in which threats will continue to occur at a high rate in the future. The individual should therefore develop a more “anxious” disposition—showing an elevated tendency or readiness to respond to potential threat [6,22]. 

The disposition to respond to potential threat has been a useful starting point for both modelling and experimentally testing predictions concerning the factors that modulate adaptive behavioural decisions. Nesse [26,27] used signal detection theory to demonstrate the “smoke detector principle” with respect to anxiety and fear behaviour in humans. In signal detection problems, individuals set a detection threshold for establishing whether a cue is indicative of a given target or just background noise. If the cost of falsely categorizing a predator as non-threatening can be death, while the cost of briefly withdrawing is small, a low detection threshold, which generates many false negative responses (*i.e.* often withdrawing from a non-predator) is optimal. Moods and/or mood disorders may affect detection thresholds (e.g., see [22,24]). Spider fearful and phobic individuals, for example, have been found to show an enhanced tendency towards false positive identifications of spider images, even though their overall accuracy at detecting spiders shows no advantage over non-fearful participants [35,36]. These and other similar findings offer empirical support for optimization hypotheses which predict that perceived outcome costs (payoffs) and perceived threat probabilities (base rate of threat) vary according to moods and mood disorders, thereby influencing decision thresholds and thus behavioural outcomes when there is potential danger [6,23,24]. 

**Figure 1:**
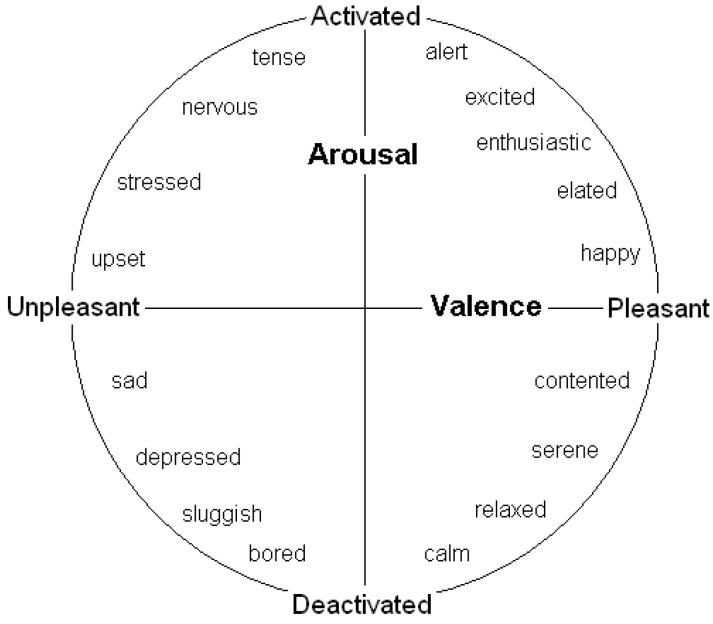
A two-dimensional representation of emotional state, governed by valence and arousal (see [11]).

Many behavioural decisions involve both potential costs (associated with danger) and potential benefits (of obtaining resources) in ambiguous situations, and the outcome may often depend on the speed of reaction. A number of authors conducting human feelings-based research have contended that the two key dimensions of affect are valence (positivity/negativity) and arousal (degree of mental alertness or activation) (e.g., see [12,13,19]). They have also found evidence that many of these emotions lie on the perimeter of a circle (see Figure 1); the full range of emotions has thus been termed an “emotion circumplex”. However, if affect can be modified by degree (e.g., moving from scared to very scared as one moves further from the origin) then it makes more sense to assume that emotions have the potential, at least, to lie across all positions in the two-dimensions rather than just on a perimeter; that is our assumption (and has been that of others such as [6]). We refer to this space as “core affect space”, following researchers (e.g., [13]) who refer to human subjective experiences that can be characterised in terms of valence and arousal (e.g., both short-term emotions and longer-term “free-floating” moods) as *core affect*. We suggest that environmental stimuli and events influence longer-term mood states and location in core affect space, which prepares the brain for decisions and the body for action. Natural selection should favour the evolution of well-prepared individuals, and here we identify why representing emotions and moods as lying in core affect space can make sense from an evolutionary perspective.

## 3. The Evolutionary Perspective: Expectation and Preparedness

While assessing a range of proposals for the evolution of general intelligence in humans, Geary, ([37], p. 22) asserted that “the central theme that cuts across generations and theories is that the core of intelligence is the ability to anticipate and predict variation and novelty and to devise strategies to cope with this novelty”. Anticipating what is likely to happen is an important function of human brains and, more than likely, the brains of other animals. This applies to many different kinds of behavioural and psychological decisions. Here, we focus on the paradigmatic problem of whether to approach a potentially fitness enhancing resource, or withdraw to avoid possible attack or other fitness-reducing harm. 

The bio-behavioural systems of mood outlined above are hypothesised to make use of prior experience to form expectations about the probabilities and values of threats and opportunities, and the individual’s current capacity to escape from or obtain them, respectively (see [6,8,23]). These systems have most often been discussed in terms of their independent functions (most notably in avoiding threats, e.g., [22,27]; but see also [6,8,17]). However, in many circumstances the integration of these two types of disposition is required, because individuals need to trade-off resource acquisition against potential costs (e.g., [38]). Whenever such tradeoffs are required, a single, bipolar dimension effectively emerges from the two-system functional view, as individuals’ behavioural decisions move towards net gains and away from net losses. In the specific case of an approach-withdrawal problem, therefore, the tendency or disposition to approach fitness enhancing resources, and to avoid or withdraw from threat, are placed in opposition to one another and the result is expressed by whether approach or withdrawal is prioritized (or which goal—achieving the resource or avoiding the threat—determines behaviour). This can be simply characterized as a single dimension, which controls the expectation of bad net outcomes at one end and the expectation of good net outcomes at the other. This parallels the dimension of valence in human circumplex models of conscious core affect when they are applied to behavioural decisions. Such a dimension may be experienced as whether, on balance, a person feels positively or negatively about a particular situation (the “how do I feel about it heuristic” [1], or the “affect heuristic” [4]).

In many situations, natural selection acts not only on the choices that are made, but on how quickly those choices are made. When decisions to approach or withdraw from ambiguous stimuli are required, it is best not only to have expectations, which facilitate those decisions but to be prepared for how quickly to make such decisions (and, physiologically, how ready to be for fast action). The benefits of such preparedness are most evident when:

(i) Good things disappear if they are not obtained in time (e.g., food in the form of prey).
(ii) Bad things remain dangerous until actively avoided (e.g., predators).

However, there are costs associated with being prepared for rapid reactions. The costs of preparedness can take more than one form:

- The *behavioural* cost of being more likely to do the wrong thing (through being too willing to respond to noisy stimuli).
- The *physiological* cost of burning resources (or storing resources in a readily useable form).
- The *attentional* cost (or opportunity cost) of preparedness increasing the probability of missing other opportunities or threats (e.g., attention for a predator decreasing the chances of detecting prey).

The costs of preparedness, together with the expected worth (benefits) of being prepared, should govern how ready to respond one should be in particular circumstances. Often, the cost of doing the wrong thing may be more significant than physiological costs. In this paper, we consider only the behavioural payoffs; *i.e.* we are interested in the speed-accuracy trade-off of decisions [39]. 

**Figure 2:**
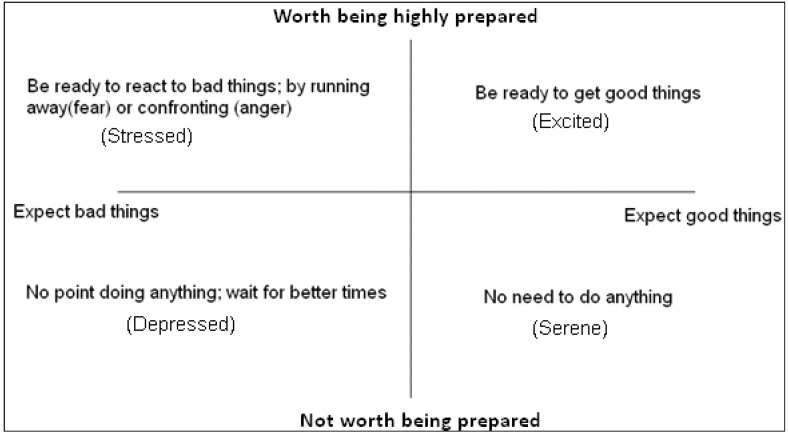
The basic functions of expectation and preparedness. Environmental cues can indicate the likely scenario (good or bad), so prepare the individual for deciding whether to approach or avoid new stimuli, and how ready to be.

The trade-off between costs and benefits of preparedness for approach or avoidance can mean that it is only worth being highly alert if rewards for being fast, or punishments for being slow, are likely to be large. Consequently, we see a state space that closely resembles two dimensional core affect space start to emerge; see Figure 2.

**Figure 3:**
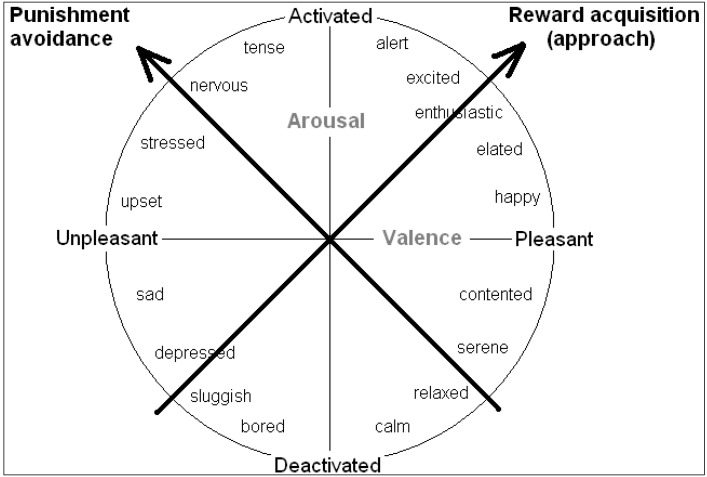
The tendency to approach or avoid a stimulus provide alternative axes of the core affect space (see [6,17,34]).

Rather than valence and arousal, it can be more worthwhile to consider inclinations for action as underlying the core affect space. Mendl *et al.* [6] endorse the idea that emotions or moods serve to guide behaviour toward “*maximizing acquisition of fitness-enhancing rewards* and *minimizing exposure to fitness-threatening punishers*”. The biobehavioural systems that subserve these two functions lie at 45 degrees to the valence and arousal axes (keeping all moods in the same position relative to each other), so the primary axes may instead be regarded as systems underlying “reward acquisition” or “punishment avoidance”, as shown in Figure 3 ([17]; see also [34]). This follows suggestions made by others for the existence of two such basic biobehavioural systems (e.g., “positive activation”, “behavioural activation” or “approach process” systems *vs.* “negative activation”, “fight flight flee”, or “avoidance process” systems [8,16,32]. 

Each sub-system may, initially, have evolved almost independently of the other. As the senses, and behavioural options, became more sophisticated, the fitness benefit of the two systems influencing each other may have increased, resulting in the sub-systems becoming almost indistinguishable from a single system, which represents a state of expectation and preparedness in the current environment. 

From an experimenter’s perspective of known payoffs and physiological measures of preparedness, the valence/arousal axes are the most obvious. However, from the decision-maker’s perspective of dispositions for action, punishment avoidance and reward acquisition can make more sense, and may reflect the activity of underlying biobehavioural systems that subserve these functions.

We thus assume that an individual’s mood state reflects their current expectations about the environment and their preparedness to act. This amounts to predispositions toward or against possible actions (more specifically, toward or away from attempting reward acquisition or punishment avoidance), governing both the probability and speed with which an option will tend to be chosen. Differences in this background expectation (and thus preparedness) should be most apparent when stimuli are novel, indistinct or ambiguous.

To show that core affect could link environmental cues and optimal decision-making, we make use of a well-known decision making model, the drift diffusion model.

## 4. The Drift Diffusion Model of Decision-Making

**Figure 4:**
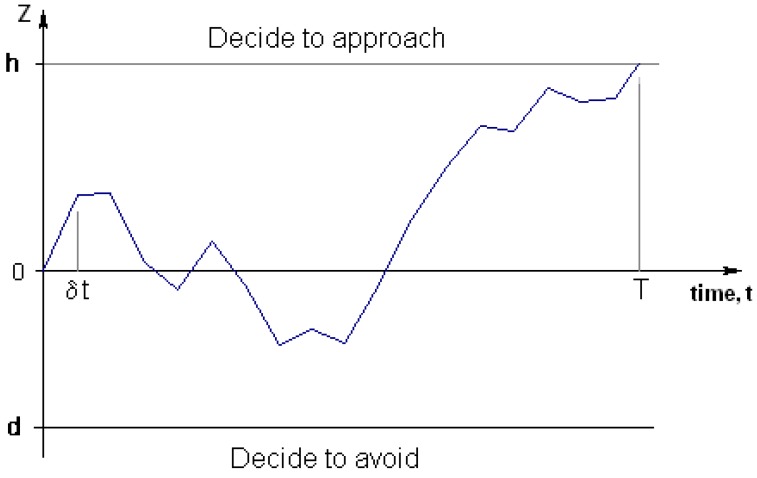
The drift diffusion model.

The Sequential Probability Ratio Test (SPRT), introduced by Wald [40], tests between two hypotheses (in this case, whether it is best to approach or withdraw). The SPRT operates by updating the relative likelihood of each hypothesis as new data arrives until deciding (with some pre-defined error probability) in favour of one of the hypotheses (see Figure 4). If data acquisition can sensibly be regarded as a stream of information, where the amount of information received per time step is normally distributed, then the information gain with respect to time equates to a Brownian motion with drift. This modelling ignores the possibility of neutral stimuli, so the drift will be positive if the novel item is good to approach, and negative if it is bad to approach. 

The two thresholds should depend on the payoffs for each choice (correct or incorrect approach or avoidance) and how those payoffs change with time. This is because the thresholds affect both the probability of making errors and the distribution of times to make a decision. 

As the time between updates decreases to zero (whilst keeping information content constant, independently sampled from a normal distribution, per unit of time), information moves according to a Brownian motion (Weiner process) with drift (positive drift if the item is good to approach).

When the payoffs in each situation are known, the optimal thresholds (which trade-off speed and accuracy to maximise expected payoff) can be calculated, and from these, the expected decision time and accuracies can be determined. This continuous time version of the SPRT is known as the drift diffusion model [41]. 

Mammals are able to integrate sensory evidence over time to increase the accuracy of their judgments ([42,43,44,45]). For example, when making a decision about direction of motion from a noisy visual stimulus, the neurons in the frontal and parietal areas, which correspond to the correct alternative gradually increase their firing rate (demonstrated in monkeys [44]). When the level of activity of the integrating neurons reaches a particular threshold, the choice is made and the corresponding action is initiated [46].

The accumulation of sensory evidence is also evident in human behavioural data. The distributions of reaction times from choice tasks between two alternatives are very well described by a drift diffusion model assuming that the difference between evidence for the two alternatives is integrated until it reaches a certain threshold [47,48,49]. The drift diffusion model is thus consistent with the neurophysiological findings [50,51] and can also account for the accuracy and reaction time of value-based choices in humans [52]. Furthermore, it has been shown that neural network models can implement the drift diffusion model for certain parameter values [41,53,54].

Under the assumed conditions (normal distributions, *etc.*), the drift diffusion model sets optimal thresholds in terms of trading off expected time to reach a decision against probability of each type of error [55]. Thus, if affective state governs the setting of thresholds in a setting where the drift diffusion model is applicable, then the core affect space can enable optimal decisions to be made under those conditions. Comparing the core affect space representation with another representation—which produced different distributions or timings of decisions—we would therefore find that individuals using the core affect space representation would be favoured by the action of natural selection.

## 5. The Model

We consider how environmental cues, indicating a threat (e.g., of a predator), or the opportunity for gaining benefits (food, sex, *etc.*) should influence decisions when the focal decision-maker is a predator to some animals whilst prey to others.

We analyse a scenario, which commences with the appearance of a novel stimulus, in the form of another animal, which will either be a predator or prey. For simplicity, we shall ignore the possibility of conspecifics or other animals, which are neither predators nor prey (although see discussion for further consideration of this). Payoffs for actions therefore relate to the probability of gaining a resource (food) or the risk of dying. We assume that information gain is normally distributed per unit of time, so the optimal decision of whether to approach or avoid accords with the drift diffusion model (Section 4), and allow the position of each boundary to be governed by core affect, which reflects the perceived state of the environment (based on previous experience).

We assume that there is a correlation between the environmental cues prior to an encounter and the probability of a newly encountered animal being a predator or prey, so the probability of the environmental condition (predator present or not) can be estimated; *p* = P(predator). On the appearance of an animal, the drift diffusion model governs how ready the individual should be to decide whether to approach or avoid it.

The optimal thresholds are governed by the probability that a predator is present, payoffs, and rates of change of payoff with time.

The time to reach the decision, *T*, can depend on which decision is reached. We let *T_ap_* represent the time to reach the decision to approach, given that the decision to approach is reached first. Similarly, let *T_av_* represent the time to reach the decision to avoid, given that the decision to avoid is reached first.

The optimal decision boundaries depend on the expected payoffs, which in turn depend on the value of the individual’s life. An individual in good condition should be less willing to risk its life for a unit of food than an individual with a greater need (though in good condition, a particular action may be less risky). The value of life is generally termed reproductive value (see [38,56]), which reflects how successful the individual is likely to be at reproducing in the future. We denote reproductive value by *V*, which will depend on the physical state of the individual; we assume that it will increase with reserves; how the reproductive value changes following a decision will depend on the current circumstances and the decision that the individual makes. Table 1 shows the expected payoff for each action, dependent on the current circumstance, which the focal individual can only estimate.

**Table 1:** Payoff matrix: value of life following a decision depends on the situation, chosen behaviour, and time to decide on that behaviour/action.

	Decide to approach	Decide to avoid
**Prey**	*V + C*(*T_ap_*)	*V*
**Predator**	0	*S*(*T_av_*)*V*

We assume that the probability of survival, *S*(*T_av_*), decreases with time to reach the decision, *T_av_*., according to S(*T_av_*) = e^−θTav^ where θ is the speed of the predator. Similarly, the expected reward for capturing a prey, *C,* decreases with *T_ap_* according to *C*(*T_ap_*) = *c*e^−λTap^ where *c* represents the size (value) of prey and λ represents the speed of prey.

In Appendix A, we show how the optimal thresholds of the drift diffusion model can be calculated. *i.e.*, we find the boundary values, *d* (avoid) and *h* (approach), which maximise E(reward|*d*,*h*).

Next, we map these thresholds to positions in the core affect space. The valence and arousal measures are inferred from the boundary values *d* (avoid) and *h* (approach) in a one-to-one manner. Conceptually, we would like to regard the reciprocal of *d* (the distance to the avoidance boundary) as the position on the punishment avoidance axis, and the reciprocal of *h* (the distance to the approach boundary) as the position on the reward acquisition axis (see Figure 3); thus, the smaller the distance to that threshold (for approach or avoid), the greater the tendency to take that action (of approach or avoid). However, to bound values on each emotional scale between 0 and 1, we use:

Position on approach axis, *x* = 2/(1 + *h*) − 1. Position on avoidance axis, *y* = 2/(1 − *d*) − 1.

These coordinates are rotated to find the valence and arousal values:

Valence = *x* cos(π/4) − *y* sin(π/4). Arousal = *x* sin(π/4) + *y* cos(π/4). 

Despite the scenario depending on many factors (*p*, λ, θ, *c*, *v*), the above process, thus, reduces to a two-parameter problem of valence and arousal. Correct setting of these values (as above) corresponds to optimal settings for the decision problem.

## 6. Results

**Figure 5:**
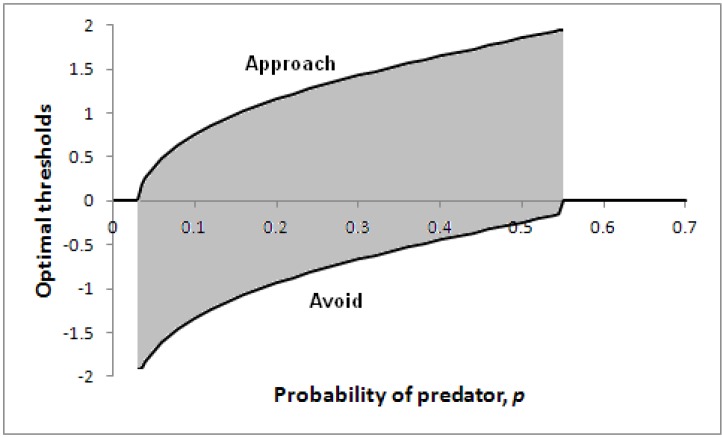
Optimal thresholds for approach (upper line) and avoidance (lower line) as a function of *p* in a particular scenario.

Figure 5 illustrates how optimal thresholds vary according to the probability of a predator being present, *p*. Note that for a sufficiently high probability of predator, the threshold for avoidance (the lower line) reduces to zero, so the individual will immediately take evasive action. In addition, if the probability of predator is extremely low, the individual will make an immediate decision (to approach).

Parameters: *V* = 5, *c* = 1, θ = 0.2, λ = 0.2. A decision only needs to be made for 0.03 < *p* < 0.55 (the shaded region); with less risk it is better to approach immediately before the likely prey has time to escape; with greater risk, it is better to avoid immediately.

**Figure 6:**
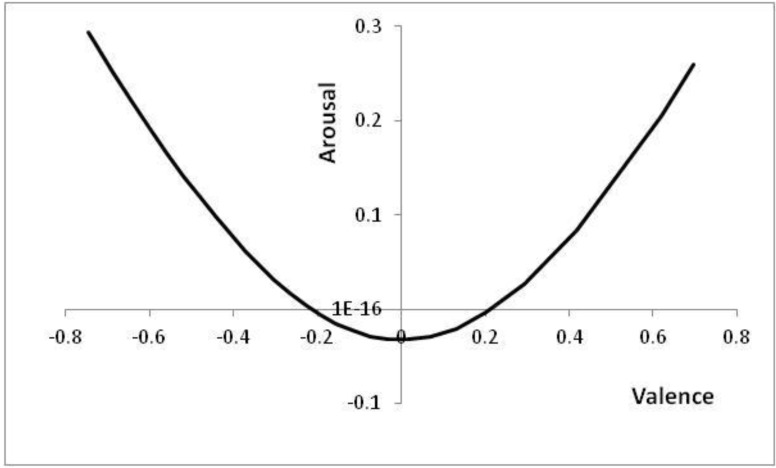
Positions reached in core affect space as *p* is altered.

Figure 6 shows the positions in core affect space that correspond to the thresholds of Figure 5, with positions to the top left of Figure 6 (high arousal and negative valence) corresponding to high *p* values (high risk of a predator being present). 

Parameters: *V* = 5, *c* = 1, θ = 0.2, λ = 0.2; *p* ∊ [0.03, 0.55] with the right-most point corresponding to the lowest risk of a predator being present (*p* = 0.03). For *p* < 0.03, the decision to approach is already made; for *p* > 0.55, the decision to avoid is already made.

Note that at either end of the line in Figure 6, the decision has already effectively been taken; for extreme *p* values, it is better to have an automatic reaction than to wait for further information (cf. [57]).

**Figure 7:**
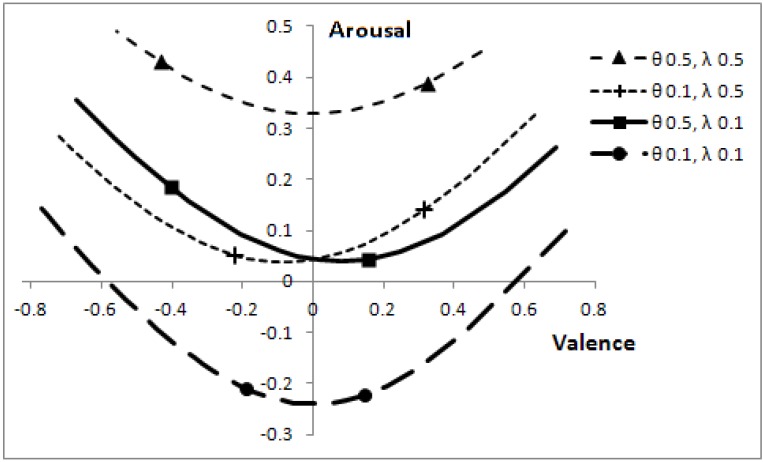
Effect of predator and prey speeds on position in core affect space, for the range of *p* values where a decision is not automatic.

Altering the speeds of predators, θ, and prey, λ, affects the optimal boundaries. The corresponding positions, which can be reached in core affect space (by varying *p*), are shown in Figure 7.

The markers on each line correspond to *p* = 0.1 (right-most markers) and *p* = 0.3. When the other animals are slow, the focal individual has low arousal and changes to the probability a predator being present have relatively less effect on valence. Parameters: *V* = 5, *c* = 1.

The length of the lines in Figure 7 can easily mislead one into thinking that when animals move at low speed (θ = 0.1, λ = 0.1), then a change in the probability that a predator is present, *p*, has a greater effect on valence than when animals move at a greater speed. The effect is, as one might expect, quite the reverse, as indicated by the line markers. When other animals are slow, a change in *p* has relatively less effect on valence than when animals are fast. The overall length of the lines is indicative of the range of *p* values over which it is sensible to make decisions (rather than react automatically to the presence of another animal); with low speed animals, the focal individual can afford to make better-informed decisions.

**Figure 8:**
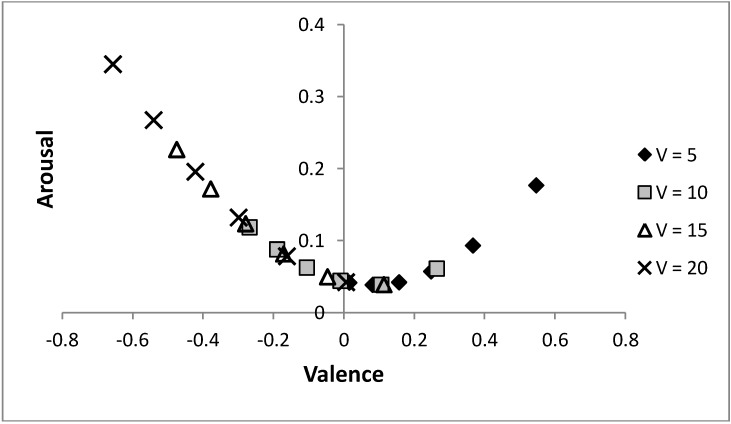
Altering the value of life and the risk of predation can produce the same moods state.

Figure 8 shows that by varying the probability of a predator, the same arc through core affect space is obtained for different values of life, *V*, only shifted (along that arc) to have more positive moods when individuals are in worse condition. Initially, it may appear counter-intuitive that the individual is in a more positively valenced state when their condition is decreased, but under such circumstances, as there is less to lose, it is adaptive to be more ready to approach novel stimuli (which is achieved by holding more positive emotions). The figure again shows that increasing risk of predation is linked to a more negatively valenced affective state and a tendency to avoid a stimulus, as predicted by models that hypothesise that affective state is influenced by previous experience of the relative rewarding or punishing nature of an environment [6,23]. The model also illustrates how perceived probability of danger and current body condition (*i.e.*, value of life, *V*) can combine to influence mood and decision-making.

As the value of life decreases, the same mood is achieved at a lower risk of predation; *i.e.*, the same arc through the core affect space is achieved for each value of life as *p* is altered. Parameters: *c* = 1, θ = 0.5, λ = 0.1, *p* ∊ {0.04, 0.06, ..., 0.14} with *p* = 0.04 (0.14) being the most (least) positively valenced occurrence for each symbol.

## 7. Discussion

We have shown that in simple situations, which require a choice between two options, an individual’s position in core affect space (*i.e.*, mood state) can encapsulate sufficient information for optimal decision processes to be carried out. This is because the two-dimensional representation can encapsulate the decision-relevant aspects of many environmental circumstances or conditions, even when these have many dimensions (five in this case, relating to the risk of predation, speed of predators, speed of prey, size of prey, and current condition/value of life).

We have assumed that the probable state of the world governs mood state (*i.e.*, position in core affect space), so it makes sense for moods to influence decisions. With strong enough background information (and thus mood), the decision can effectively have been made before new data even arrives. As de Waal [58] puts it, “emotions potentiate actions”. Together with the potential benefit or punishment (*i.e.*, valence) associated with new stimuli, there is an advantage to being prepared but also a cost for responding too quickly. Here, we have only considered the cost in terms of decision-making errors; generally there may also be a physiological cost for being prepared for action. 

The optimality of the core affect space has been demonstrated by remapping the two threshold parameters of the drift diffusion model. Thus, it is straightforward that the core affect space representation of emotions can be said to be optimal in any situation in which the drift diffusion model is optimal. (If the decision problem could not be reduced to two parameters, then core affect space would be insufficient.) The situation need not be one of ambiguous predator/prey cues; simple uncertainty over the probability that a predator is present can be reflected by a drift diffusion model so the standard trade-off between energy and predation (e.g., [59,60]) can be related directly to the core affect representation. Future work can focus on other aspects, such as the effect of actions altering future payoffs, perhaps through feedback affecting others, such as the effect of anger on decision-making in social situations.

Both an individual’s prior experience as well as current environmental cues can potentially be reliable indicators of the current conditions. From a human psychological perspective, the present model closely parallels self-reported, current, consciously experienced affect (emotions and mood states). However, we have not addressed why emotions “feel” and whether these feelings themselves have any decision-making functions. This paper provides a functional perspective on how expectations should affect readiness for different possible actions, and how these appear to resemble felt “moods”. Whether this is merely a superficial resemblance, or one that stands up to more detailed scrutiny, remains to be investigated. 

This model indicates that when faced with the same situation, individuals with a low value of life (e.g., a starving individual) should have a greater valence than individuals with a higher value of life. Although counter-intuitive, the effect is adaptive in this representation because starving individuals have less to lose (relative to what they would gain), so should be more ready to approach novel stimuli. If circumstances (such as the risk of predators being present, *p*) were not known and could only be learned about over time, low reserves might have been reached whilst learning that the current environment is likely to be dangerous. In general a bad experience, such as having to avoid a predator, might have two effects: increase the perception that the world is currently bad, and increase the need for food. The overall change in mood (from these contrasting influences) will depend on how much the individual’s condition has changed, and how much has been learned about the risk of predation (*i.e.*, how much *V* and *p* have changed; see Figure 8).

It might be argued that rather than a state of mental (or physiological) preparedness for action, the level of “arousal” in an individual could be set by the worth of going out exploring, irrespective of any immediately novel stimuli. However, in order to explore, one needs to approach *something*, which is unknown (even if it’s just a space on the horizon). Thus, on closer inspection, such a behavioural view of arousal does not appear to conflict sharply with the approach we have chosen. Dayan [61] takes a similar line, referring to “vigour” rather than “arousal” (cf. [62]).

In situations where great fitness benefits can arise from rapid integration of sensory information with background expectations (e.g., making a decision about whether to attack or flee when a new animal appears, based on the immediate visual information of eye saccades and general circumstances), it makes sense for the background information to be stored in a concise form for use by the relevant decision “modules” which integrate this with the sensory information (e.g., the hippocampus; see [63], pp. 283–288). Here, we have shown that rather than take many variables into account, any such decision module would need just two inputs (tendency to approach and tendency to avoid) in the kind of circumstances we have considered. In more complicated situations, it may be necessary for the decision-making mechanism to have additional input parameters relating to the environment; we leave this to future papers.

## 8. Future Directions

### 8.1. Taking Account of Future Payoffs

This paper has considered only a single decision, but the value of life, V, is dependent on the future. In a social situation where choices can feed back through others to affect probabilities and payoffs in future scenarios, additional complexity would need to be introduced in relation to the possible actions for this to be modelled explicitly. To do so would require at least one extra axis, to account for the longer-term effects; this may also allow the differential effects of states that cannot be distinguished completely by two dimensions (e.g., fear and anger) to be considered. It may also be interesting to consider a sequence of decisions (rather than just one, as done in this paper) to predict the effects of reserve levels; this would also allow the value of life to emerge from the model, rather than be input.

### 8.2. Timescales of Emotional Change

This paper has assumed that prior experiences determine a single position in core affect space, and that this position governs boundaries, which do not move during the decision-making process. Alternatively, it could be argued that new information should transfer instantaneously into movement in core affect space, with a decision being reached when one of the corresponding threshold boundaries is reduced to zero, but such movement would then relate only to the current decision. However, the rate of movement in core affect space may be governed by a similar, longer-term representation if long-term states can affect short-term core affect over time. 

### 8.3. Links with Learning

Extending the model to incorporate learning may help to establish the degree to which core affect (and life-history strategies) should be learned, as opposed to genetically set in relation to stimuli. Rates of change of value with time (from appearance of novel stimuli) should affect the level of arousal. For instance, individuals growing up in environments where there is little need for speed may display a different amount of movement on the arousal axis. Niv, Joel, and Dayan [64] discuss this in the context of goal-directed and habitual behaviour of rats with different motivational states. At a more detailed level, it may be possible to consider how learning and physiology are related through the effects of dopamine and serotonin (see [33]). Learning about a benign environment with few predators and high food abundance will decrease the individual’s perception of the probability of danger, resulting in a more positively valenced state (Figure 6), but at the same time, it is likely to increase its body condition and this may actually lead to a decreased tendency to respond to stimuli, driven by a lower relative valuation of the possible reward.

### 8.4. Multiple Choices

The current model has only two possible outcomes. In many situations, there are more than two choices; e.g., the choice to approach, withdraw, or ignore (treat the novel stimulus as neither beneficial nor harmful). However, “the case of more than two options is surprisingly complicated” as Dayan and Daw ([65], p. 446) put it whilst discussing the sequential probability ratio test component of the drift diffusion model. Consequently, it is not clear how best to incorporate such choices into the existing model. 

### 8.5. Physiological Costs

One state of preparedness may be limiting under certain circumstances, because an individual may sometimes need to be very prepared to run away, but not to approach rapidly. Thus, behavioural and decision-theoretic measurements of arousal may differ, raising the question of how physiology (such as the level of glucocorticoids) is linked with decision-making parameters, and the extent to which physiological costs should influence the decision-making system.

### 8.6. Comparisons across Taxa

Herbivores, which have little need of rapid approach but need to be wary of predators, may have little need for an approach threshold, whereas top predators may have little in the way of an avoidance threshold. Modelling these decision-makers, in conjunction with a comparison of decision-making apparatus (*i.e.*, brain structure), may provide useful insights into the operation of core affective structure in different types of animal.

In summary, approaches using core affect space may be useful in developing theoretical links between stimuli, drives, emotions, physiology, decision making, and behaviour. In this case, we have demonstrated conditions under which moods, as represented by two parameters, are optimal. How to modify this assumption of optimality in a meaningful way, to provide deeper understanding of the biological phenomena, remains an open question.

Our aim in this paper has not been to show that core affect space is a realistic representation of emotions, still less that the brain actually represents emotions in this dual-axis manner—it is also possible that emotions are the result of a complex interplay between different parts (or modules) of the brain (see [66]). Rather, our aim has been to show that core affect space is a sensible representation of emotional mood states (from a general evolutionary perspective) in many scenarios. This is the case because the representation makes sense from the perspective of optimal performance. As such, we suggest that linking the core affect representation with the drift diffusion model in the above manner may enable various aspects of emotions and moods to be studied in a useful manner.

## Appendix A: Calculating Optimal Boundaries in the Predator/Prey Model

The time to reach the decision, *T*, can depend on which decision is reached. We let *T_ap_* represent the time to reach the decision to approach, given that the decision to approach is reached first. Similarly, let *T_av_* represent the time to reach the decision to avoid, given that the decision to avoid is reached first. The payoffs are shown in Table 1.

Probability of survival, *S*(*T_av_*), decreases with time to reach the decision, *T_av_*. Assume S(*T_av_*) = e^−θ*Tav*^. Note *C* must decrease with *T_ap_* or the decision to approach will never be reached. Assume *C*(*T_ap_*) = ce^−^^*λTap*^.

We assume the probability of predator is *p*, so the probability of prey is (1-*p*). The expected value, given boundaries *d* and *h*, is

E(reward| *d*,*h*) =

(1)

Approaching a predator gives zero reward, and avoiding prey simply gives the current value of the animal’s life, *V*, so this reduces to:

E(reward| *d*,*h*) =

(2)

The handbook of Brownian motion ([67], p. 233) gives that the probability of avoiding predation in time dt at time t is:

(3)

where μ is the mean drift of information with time and (p.451):

(4)

Note that the equations are assuming that information is typically gained at the same rate, μ, whether the animal is a predator or prey. We assume that drift in information is positive (μ) when the animal is prey and negative (-μ) when a predator. Thus,

E(reward| *d*,*h*) =

(5)

Rearranging, we obtain:

E(reward|*d*,*h*) =

(6)

E(reward|*a*,*b*) =

(7)

In this case, the starting point, x, is zero. Approximating the infinite sums by discarding terms with k outside a given range, of [–10, 10], we obtain:

E(reward|*a*,*b*) ≈

(8)

We perform the integration numerically, using a time step of 0.001s and integrating over 100 seconds using the midpoint rule.

By finding the boundary values, *d* and *h*, which maximise E(reward|*d*,*h*), we therefore identify the optimal thresholds which correspond to the specified environment (*i.e.*, given *p*, *μ*, λ, θ, *c*, V).

Next, we map these thresholds to positions in core affect space.

Here, we use a very simple system. Conceptually, we would like to regard the reciprocal of *a* (the distance to the avoidance boundary) as the position on the punishment avoidance axis, and the reciprocal of *b* (the distance to the approach boundary) as the position on the reward acquisition axis (see Figure 3). However, to bound values on each emotional scale between 0 and 1, we use:

Position on approach axis, *x* = 2/(1 + *h*) – 1. Position on avoidance axis, *y* = 2/(1 − *d*) − 1.

These coordinates are then rotated to find the valence and arousal values:

Valence = *x* cos(π/4) − *y* sin(π/4). Arousal = *x* sin(π/4) + *y* cos(π/4).

## Isomorphic Mapping

Note that the system we have assumed provides a one-to-one mapping between emotions and decision boundaries (assuming V, λ, θ, *c* are fixed). Starting from the emotions: a given position in core affect space maps directly to a distinct position in the approach/avoidance space, (*x*, *y*). From *x* we can calculate *h*, and from *y*, *d*. Similarly, it is clearly 1-to-1 going from boundaries *d* and *h* to valence and arousal.

## References

[1] Schwarz N., Clore G.L. (1983). Mood, Misattribution, and judgments of well-being—informative and directive functions of affective states. J. Pers. Soc. Psychol..

[2] Gasper K., Clore G.L. (1998). The persistent use of negative affect by anxious individuals to estimate risk. J. Pers. Soc. Psychol..

[3] Forgas J.P. (1995). Mood and judgment—the affect infusion model (AIM). Psychol. Bull..

[4] Slovic P., Finucane M.L., Peters E., MacGregor D.G. (2007). The affect heuristic. Eur. J. Res..

[5] Paul E.S., Harding E.J., Mendl M. (2005). Measuring emotional processes in animals: The utility of a cognitive approach. Neurosci. Biobehav. R..

[6] Mendl M., Burman O.H.P., Paul E.S. (2010). An integrative and functional framework for the study of animal emotion and mood. Proc. Biol. Sci..

[7] Bradley M.M., Lang P.J. (1994). Measuring emotion—the self-assessment mannequin and the semantic differential. J. Behav.Ther. Exp. Psychiatr..

[8] Carver C.S. (2001). Affect and the functional bases of behavior: On the dimensional structure of affective experience. Pers. Soc. Psychol. Rev..

[9] Colibazzi T., Posner J., Wang Z.S., Gorman D., Gerber A., Yu S., Zhu H.T., Kangarlu A., Duan Y.S., Russell J.A. (2010). Neural systems subserving valence and arousal during the experience of induced emotions. Emotion.

[10] Larsen R.J.D., Diener E., Clark M.S. (1992). Promises and problems with the circumplex model of emotion. Review of Personality and Social Psychology: Emotion.

[11] Posner J., Russell J.A., Peterson B.S. (2005). The circumplex model of affect: An integrative approach to affective neuroscience, cognitive development, and psychopathology. Dev. Psychopathol..

[12] Russell J.A. (1980). A circumplex model of affect. J. Pers. Soc. Psychol..

[13] Russell J.A. (2003). Core affect and the psychological construction of emotion. Psychol. Rev..

[14] Russell J.A., Barrett L.F. (1999). Core affect, prototypical emotional episodes, and other things called Emotion: Dissecting the elephant. J. Pers. Soc. Psychol..

[15] Thayer R.E. (1989). The Biopsychology of Mood and Activation.

[16] Watson D., Wiese D., Vaidya J., Tellegen A. (1999). The two general activation systems of affect: Structural findings, evolutionary considerations, and psychobiological evidence. J. Pers. Soc. Psychol..

[17] Watson D., Tellegen A. (1985). Toward a consensual structure of mood. Psychol. Bull..

[18] Yik M.S.M., Russell J.A., Barrett L.F. (1999). Structure of self-reported current affect: Integration and beyond. J. Pers. Soc. Psychol..

[19] Yik M., Russell J.A., Steiger J.H. (2011). A 12-point circumplex structure of core affect. Emotion.

[20] Frijda N.H. (1986). The Emotions. Studies in Emotion and Social Interaction.

[21] Ekman P. (1992). An Argument for Basic Emotions. Cognit. Emot..

[22] Bateson M., Brilot B., Nettle D. (2011). Anxiety: An evolutionary approach. Can. J. Psychiat..

[23] Nettle D., Bateson M. (2012). The evolutionary origins of mood and its disorders. Curr. Biol..

[24] Paul E.S., Cuthill I., Kuroso G., Norton V., Woodgate J., Mendl M. (2011). Mood and the speed of decisions about anticipated resources and hazards. Evol. Hum. Behav..

[25] Blanchette I., Richards A. (2010). The influence of affect on higher level cognition: A review of research on interpretation, judgement, decision making and reasoning. Cognit. Emot..

[26] Nesse R.M. (2001). The smoke detector principle—Natural selection and the regulation of defensive responses. Ann. NY Acad. Sci..

[27] Nesse R.M. (2005). Natural selection and the regulation of defenses—A signal detection analysis of the smoke detector principle. Evol. Hum. Behav..

[28] Mendl M., Paul E.S., Chittka L. (2011). Animal behaviour: Emotion in invertebrates?. Curr. Biol..

[29] Rolls E.T. (2005). Emotion Explained (Series in Affective Science).

[30] Panksepp J. (2011). The basic emotional circuits of mammalian brains: Do animals have affective lives?. Neurosci. Biobehav. Rev..

[31] Carver C.S., Scheier M.F. (1990). Origins and functions of positive and negative affect—a control-process view. Psychol. Rev..

[32] Gray J.A., Davidson P.E.R.J. (1994). Three fundamental emotion systems. The Nature of Emotion.

[33] Boureau Y.L., Dayan P. (2011). Opponency revisited: Competition and cooperation between dopamine and serotonin. Neuropsychopharmacology.

[34] Burgdorf J., Panksepp J. (2006). The neurobiology of positive emotions. Neurosci. Biobehav. Rev..

[35] Becker E.S., Rinck M. (2004). Sensitivity and response bias in fear of spiders. Cognit. Emot..

[36] Wiens S., Peira N., Golkar A., Ohman A. (2008). Recognizing masked threat: Fear betrays, but disgust you can trust. Emotion.

[37] Geary D.C., Platek S.M., Shackelford T.K. (2009). The evolution of general fluid intelligence. Foundations in Evolutionary Cognitive Neuroscience.

[38] Houston A.I., McNamara J.M. (1999). Models of Adaptive Behaviour.

[39] Chittka L., Skorupski P., Raine N.E. (2009). Speed-accuracy tradeoffs in animal decision making. Trends Ecol. Evol..

[40] Wald A. (1945). Sequential tests of statistical hypotheses. Ann. Math. Stat..

[41] Bogacz R., Brown E., Moehlis J., Holmes P., Cohen J.D. (2006). The physics of optimal decision making: A formal analysis of models of performance in two-alternative forced-choice tasks. Psychol. Rev..

[42] Cisek P., Kalaska J.F. (2005). Neural correlates of reaching decisions in dorsal premotor cortex: Specification of multiple direction choices and final selection of action. Neuron.

[43] Schall J.D. (2001). Neural basis of deciding, choosing and acting. Nat. Rev. Neurosci..

[44] Shadlen M.N., Newsome W.T. (2001). Neural basis of a perceptual decision in the parietal cortex (area LIP) of the rhesus monkey. J. Neurophysiol..

[45] Yang T., Shadlen M.N. (2007). Probabilistic reasoning by neurons. Nature.

[46] Roitman J.D., Shadlen M.N. (2002). Response of neurons in the lateral intraparietal area during a combined visual discrimination reaction time task. J. Neurosci..

[47] Laming D.R.J. (1968). Information Theory of Choice-Reaction Times.

[48] Ratcliff R. (1978). A theory of memory retrieval. Psychol. Rev..

[49] Ratcliff R., Smith P.L. (2004). A comparison of sequential sampling models for two-choice reaction time. Psychol. Rev..

[50] Smith P.L., Ratcliff R. (2004). Psychology and neurobiology of simple decisions. Trends Neurosci..

[51] Bogacz R. (2007). Optimal decision-making theories: Linking neurobiology with behaviour. Trends Cognit. Sci..

[52] Milosavljevic M., Malmaud J., Huth A., Koch C., Rangel A. (2010). The Drift Diffusion Model can account for the accuracy and reaction time of value-based choices under high and low time pressure. Judgm. Decis. Mak..

[53] Wong K.F., Wang X.J. (2006). A recurrent network mechanism of time integration in perceptual decisions. J. Neurosci..

[54] Usher M., McClelland J.L. (2001). The time course of perceptual choice: The leaky, competing accumulator model. Psychol. Rev..

[55] Wald A., Wolfowitz J. (1948). Optimum character of the sequential probability ratio test. Ann. Math. Stati..

[56] Fisher R.A. (1930). The Genetical theory of Natural Selection.

[57] Trimmer P.C., Houston A.I., Marshall J.A.R., Bogacz R., Paul E.S., Mendl M.T., McNamara J.M. (2008). Mammalian choices: Combining fast-but-inaccurate and slow-but-accurate decision-making systems. Proc. Biol. Sci..

[58] De Waal F.B.M. (2011). What is an animal emotion?. Ann. NY. Acad. Sci..

[59] Houston A.I., Mcnamara J.M., Hutchinson J.M.C. (1993). General results concerning the trade-off between gaining energy and avoiding predation. Philos. T. Roy. Soc. B.

[60] Lima S.L., Dill L.M. (1990). Behavioral decisions made under the risk of predation—a review and prospectus. Can. J. Zoolog..

[61] Dayan P. (2012). Instrumental vigour in punishment and reward. Eur. J. Neurosci..

[62] Niv Y., Daw N.D., Joel D., Dayan P. (2007). Tonic dopamine: Opportunity costs and the control of response vigor. Psychopharmacology.

[63] Buzsáki G. (2006). Rhythms of The Brain.

[64] Niv Y., Joel D., Dayan P. (2006). A normative perspective on motivation. Trends Cognit. Sci..

[65] Dayan P., Daw N.D. (2008). Decision theory, reinforcement learning, and the brain. Cogn. Affect. Behav. Ne..

[66] Phan K.L., Wager T., Taylor S.F., Liberzon I. (2002). Functional neuroanatomy of emotion: A meta-analysis of emotion activation studies in PET and fMRI. Neuroimage.

[67] Borodin A.N., Salminen P. (1996). Handbook of Brownian Motion—Facts and Formulae (Probability and Its Applications).

